# “My Bitch Is Empty!” an Overview of the Preconceptional Causes of Infertility in Dogs

**DOI:** 10.3390/vetsci12070663

**Published:** 2025-07-12

**Authors:** Juliette Roos-Pichenot, Maja Zakošek Pipan

**Affiliations:** 1 ANIREPRO, 226 Boulevard Pommery, 51100 Reims, France; roos.juliette@gmail.com; 2 Clinic for Reproduction and Large Animals, Veterinary Faculty, University of Ljubljana, Gerbičeva 60, 1000 Ljubljana, Slovenia

**Keywords:** infertility, estrus cycle, breeding soundness examination, breeding management, semen

## Abstract

Infertility is a complex and common problem in reproductive medicine consultations. Three factors must be examined during the preconception phase: breeding management, the fertility of the bitch, and the fertility of the stud dog. Among these factors, improper breeding management remains the main cause of reproductive failure, with accurate recognition of ovulation being crucial for successful mating. Artificial insemination allows for a thorough evaluation of semen quality compared to natural mating. In addition, genetic selection, nutritional factors, and reproductive health management can either impair or improve the fertility of females and males. Idiopathic infertility can occur in bitches, but it is important to rule out other possible causes first. In bitches with irregular estrus cycles, ovarian dysfunction and endocrine imbalances should be investigated. In bitches with regular cycles, uterine disorders such as cystic endometrial hyperplasia, endometritis or congenital anomalies may be the cause. Both mating-related and chronic endometritis are recognized as contributing factors to infertility. Infectious agents, particularly *Brucella* spp. and *Mycoplasma* spp., should also be evaluated, although interpretation of *Mycoplasma* test results requires caution. In males presenting with poor semen quality, potential causes include infectious diseases (with brucellosis always requiring exclusion), hormonal imbalances, and the impact of exogenous treatments. The article underscores the critical role of comprehensive diagnostic protocols, proactive health surveillance, and data-driven breeding strategies in systematically addressing this multifaceted challenge.

## Simple Summary

This article examines infertility in dogs, focusing on three main areas: breeding management, female fertility, and male fertility. The most common cause of failed conception is poor timing of ovulation, which can be improved by modern techniques such as progesterone testing and vaginal cytology. Artificial insemination increases the chances of successful breeding, as it enables precise timing of insemination—one of the most important determinants of fertility. It also offers the possibility of assessing sperm quality and thus recognizing or ruling out infertility of the male factor. Fertility in both sexes is also influenced by genetics, diet, and environmental stress. In females, irregular estrus cycles may indicate ovarian or hormonal disorders, while normal cycles with infertility often indicate uterine conditions such as chronic inflammation or congenital problems that can be detected with imaging. In males, fertility problems are often due to infections, hormonal imbalances, or medication. Modern diagnostic procedures, such as the anti-Müllerian hormone test, offer new hope for better reproductive management. This article emphasizes the importance of thorough diagnosis, proactive health monitoring, and evidence-based breeding to effectively treat this complex issue.

## 1. Introduction

According to the World Health Organization, infertility can be defined in humans as “a failure to achieve a pregnancy after 12 months or more of regular unprotected sexual intercourse”. It can be due to male factors, female factors, a combination of both factors, or can also be unexplained and would affect one in six people (proportion of total infertility in human couples) [1].

In dogs, infertility affects a smaller proportion of individuals. However, it is a major issue for breeders. True infertility in the bitch, which could be defined by the authors as a failure to conceive after using the means of assisted reproduction currently available in this species and using various males whose fertility has been recently proven, is a fairly rare condition. Surprisingly, despite numerous studies that have looked at the causes and treatment protocols of infertility, there is still no accurate quantitative data on the prevalence of infertility [2,3,4]. A study conducted in France with 27,221 bitches (248 different pure breeds) and 45,913 heats showed an expected normal whelping rate of 81.9% [5].

Conception failure refers to the inability of the bitch to produce viable offspring and can be separated into two parts:

1. Preconceptional causes, meaning that the bitch cannot become pregnant. These causes can be divided into three main categories: breeding management-related causes of failure and female and male causes of preconceptional failure. These are the causes of infertility on which this review will focus;
2. Failure to maintain a pregnancy. These causes of infertility have been described in detail in a recent review [6].

After years of research and dozens of publications, many breeders are still convinced that the female is the main cause of breeding failure. However, ovulation detection, mating, or insemination management are the keys to successful breeding. This means that breeding management is still the main cause of failure, with the incidence of breeding failures ranging from 40% to over 50%, according to several authors [7,8]. Selection and genetics as parts of breeding programs can also have a negative impact on fertility and have also been evaluated in this review.

Concerning the bitch, it can be challenging to categorize the different causes of infertility. This review adopts a clinical perspective, analyzing the issue based on the phases of the reproductive cycle. The initial consideration is whether the bitch exhibits regular or irregular estrous periods. Recent studies have highlighted the significance of the uterine environment in canine fertility, underscoring its impact on reproductive success [9,10,11,12]. This topic will be a focal point of this discussion, particularly for bitches with regular cycles and well-managed breeding practices. Infectious diseases will also be assessed in bitches with regular cycles presenting with infertility. Conversely, in bitches with irregular estrus cycles, endocrine and ovarian alterations will be explored.

Finally, male fertility must always be evaluated as a potential preconceptional factor contributing to pregnancy failure. Addressing male infertility can be particularly frustrating. While semen collection and analysis are relatively straightforward, identifying the root cause of infertility and establishing an effective treatment plan often present significant challenges. In many cases, unresolved infertility signals the premature end of a stud dog’s breeding career. This review will present the primary causes of infertility in stud dogs, including ejaculation disorders and defects in semen quality, along with potential management strategies.

## 2. Breeding Management as a Preconceptional Cause of Infertility

Infertility consultations in veterinary practice are predominantly sought for purebred animals, underscoring the critical role of effective breeding management as a primary determinant of success. The “purebred” status often introduces a unique set of challenges that must be carefully addressed. These challenges may include geographical distance between the female (bitch) and the selected male (stud dog), the age of the animals at the time of initial mating (as some breeders opt to delay reproduction until after the animal’s competitive or aesthetic career), and the elevated genetic value of the animals, which is frequently accompanied by heightened owner expectations. These factors collectively contribute to the complexity of managing infertility in purebred animals, necessitating a scientifically informed and meticulously planned approach to breeding.

In this section, we look at the various preconceptual causes of infertility in the bitch, with a focus on breeding management. Topics covered include the approach to the initial consultation, the influence of age, genetic background, selection, and the role of nutrition. In addition, strategies for optimizing natural mating and artificial insemination will be discussed, including ovulation timing, scheduling, and procedure management. Practical guidelines for dealing with these factors in clinical practice are also provided.

### 2.1. How to Start the Consultation

When a bitch is presented for “infertility”, the consultation should start with a thorough examination of the commemoratives and anamnesis.

First, general questions should be asked to obtain information about the general environment of the animals. This includes the following:

○ Housing conditions and social structure of conspecifics, including the way the animals are grouped, their spatial distribution, and social interactions in the environment;
○ Vaccination status (herpes virus, core and uncore vaccines, whether the bitch was regularly vaccinated);
○ Deworming protocol (including specific considerations for pregnant animals and neonates);
○ Feeding process (what kind of food, how is it delivered and preserved; bigger attention should be given to BARF, with any supplements);
○ Travels/Exhibitions (in what countries; whether the animals had any kind of contact with other animals; how the animals are reintroduced in the breeding facility, especially respecting the quarantine period);
○ Medical and surgical history and possible ongoing treatments.

The second part of commemoratives includes the general reproductive history: age of the animals (females and males used), reproduction history of the lineage (sisters, mother, aunts, etc.), status of the bitch (primiparous versus multiparous), and description of previous breeding (number of litters, litter size, types of mating and whelping, possible difficulties during gestation or for the neonates).

Finally, the proper reproductive anamnesis of the bitch justifying the consultation should be studied. Here are some examples of questions that could be asked:

○ Does the male have known fertility?
○ Did the male mate before?
○ Did the dogs stay attached?
○ How many matings were made and over what period (for example: two matings in 3 days, one protrusion per day for 10 days)?
○ Did the bitch have a heat follow-up?
○ When was the pregnancy diagnosis conducted (as soon as 18 to 20 days post-ovulation or later)?

All those questions will help the veterinarian to have a first orientation of what could be proposed to achieve successful breeding by identifying the different possible moments of the breeding program that could have improper management.

### 2.2. Age of the Animals

The impact of age on fertility affects both males and females in different parameters.

In contrast to humans where menopause marks a definitive end to reproductive capacity, no such phenomenon is described in bitches. However, while some animals conceive even at advanced ages, fertility naturally declines over time. True infertility may never be reached, but each estrous period in older females poses significant reproductive risks, as pregnancy may become dangerous. One of the main reasons described for this decrease in fertility in aging bitches is the development of a cystic endometrial hyperplasia (CEH). Although primarily considered a post-conceptional cause of pregnancy failure, its impact on reproductive success warrants attention during preconception evaluations, since its diagnosis should be made before conception. Briefly, the development of a CEH is correlated with repeated exposure to sexual hormones, estrogens and progesterone, which can be endogenously produced or exogenously administered [13,14,15]. The uterine environment of bitches affected by CEH is not suitable for embryo implantation. Moreover, even though pyometra can develop without the occurrence of CEH, it has been shown that CEH facilitates the development of uterine infection that may result in early pregnancy loss [16,17]. Diagnosis of CEH at first genital examination of an infertile bitch is an important prognostic factor, often indicating poor reproductive potential due to limited treatment options. The idea of the treatment is to give the endometrium some time to generate by inducing a prolonged anestrus. As the use of progestins is not an option, authors have proposed the use of mibolerone, which is an androgen-receptor agonist [18]. This medicine should be used with caution as it is contraindicated during pregnancy and may lead to masculinization of the fetus. A pregnancy diagnosis should always be carried out before prescribing.

In a study conducted by Moxon et al. in 2016 involving 240 bitches of various pure and mixed breeds, it was found that the incidence of CEH was less than 7% in animals younger than 2 years old and more than 60% in animals older than 6 years [12]. CEH is, therefore, a degenerative disease found in higher proportion in ageing bitches.

The age of the dam has also been shown to have a negative impact on litter size, and in a large-scale Swedish study, this had a stronger effect in larger breeds [19]. Interestingly, a recent study focusing on German Shepherd dogs reported that the ages of both the bitch and the male influenced the sex ratio of the litter. However, it is important to note that these findings are specific to this breed and require further validation across different breeds to confirm their general applicability [20].

In males, age appears to have a smaller impact on fertility. Stud dogs can maintain their fertility into old age, which does not mean that the collection of old males is always harmless (orthopedic or cardiac diseases, for example, may put the animal at risk during collection). Even if the effects are less obvious, they cannot be ruled out. In several studies, with the exception of that of Goericke-Pesch & Failing, 2013, age had no effect on semen volume, sperm concentration, and total sperm production [21,22,23]. Sperm mobility, morphology, and viability, on the other hand, are negatively impacted by age [21,23,24,25]. Hesser et al. conducted an interesting study comparing three groups of Labrador retriever dogs (young (n = 21), middle-aged (n = 13), and senior (n = 5)) with the results of fresh and chilled semen. In these groups of dogs, the senior dogs showed a lower progressive motility in chilled semen compared to all other samples, and velocity also decreased with age, with greater effects in chilled semen. Morphology was also negatively affected by age. However, age had no effect on fertility and fecundity [24].

The chromatin structure of spermatozoa also appears to be negatively influenced by age [21,24]. Although, to the author’s knowledge, no study to date has shown a statistical difference between semen quality regarding chromatin integrity, taking age into account between fresh and frozen semen, it can be assumed that age may have a stronger negative influence on post-thawed semen quality. Since breeders always prioritize the show and working career of their stud dogs before thinking about DNA preservation, this would be an additional argument to insist that cryopreservation should be performed in younger animals.

### 2.3. Genetics and Selection

Working with breeds naturally means having to deal with the genetic poverty and risk of inbreeding. Inbreeding is more or less present in the different breeds and can have a negative impact on a dog’s health [26]. Concerning reproduction, inbreeding has been associated with a reduction in litter size [27]. However, this effect does not appear to be consistent across all breeds. For example, in Irish Wolfhound analyses of maternal and paternal inbreeding coefficients over 5 to 30 generations, there was no indication of a significant impact on fertility [28]. On the contrary, a study on a cohort of 93 Golden Retrievers found a negative correlation between genomic inbreeding coefficients and both the number of live-born puppies and the conception rate of bitches, although the latter association did not reach statistical significance [29].

Much work remains to be performed in exploring the relationship between inbreeding and fertility. Nevertheless, many breeders already consider the inbreeding coefficient when selecting ideal pairings, often driven more by health concerns than fertility.

Going further, in the context of inbreeding, some traits of fertility have been shown to be heritable. Litter size, for example, is a heritable trait as well as the neonatal survival and puppies’ weight at birth in some breeds [30].

### 2.4. Nutrition

Nutrition plays a crucial role in all physiological functions, including reproduction. One of the first indications of this is the onset of puberty, which requires a specific level of body development. Sones & Balogh (2020) referenced earlier studies demonstrating that puberty was reached in Labrador Retrievers and Beagles when they attained approximately 71% and 81% of their adult body weight, respectively [31]. An important example of the relationship between nutrition and reproductive health is the impact of obesity, which has been linked to hormonal imbalances such as hyperprolactinemia, potentially affecting luteinizing hormone (LH) production. Additionally, obesity alters leptin and insulin-like growth factor 1 (IGF-1) concentrations, influencing LH signaling to the gonads and, possibly, ovarian function [32]. Moreover, inflammatory factors secreted by fat cells (such as TNF-α) may interfere with the hypothalamic–pituitary–gonadal axis, disrupting normal hormonal regulation and reproductive function. Finally, obesity is associated with reduced conception rates and smaller litter sizes, while underweight bitches are prone to irregular estrous cycles and difficulties in achieving and/or maintaining pregnancy [31,33].

The effects of both underweight and overweight conditions on fertility, as well as their influence throughout all stages of reproduction, have been very well reviewed recently by Kim & Wakshlag (2021) and Sones & Balogh (2020) [31,34].

The key takeaway for breeders is that a bitch should maintain an ideal body condition score before entering the reproduction cycle. The recommended ideal body score falls within the range of 4 or 5 on the nine-point scale [25,31].

### 2.5. Ovulation Detection: Timing of Breeding

It is perhaps redundant to reiterate the pivotal role of ovulation determination in ensuring successful mating outcomes in canines. Nevertheless, this fundamental aspect of reproductive management remains underappreciated by some breeders, who continue to adhere to traditional practices of scheduling matings between the 12th and 14th day of the estrus cycle without considering the significant variability in estrus duration among individual bitches [35]. Such an approach often overlooks the critical window for optimal fertility, potentially compromising breeding success.

While a comprehensive review of all ovulation determination techniques is beyond the scope of this article, we aim to highlight the most recent advancements in estrus monitoring and address the frequently overlooked steps that are essential for accurate timing of mating. These steps, though often neglected by many veterinarians, are crucial for maximizing conception rates and ensuring the health and well-being of both the dam and the offspring.

Recent studies have emphasized the importance of combining multiple diagnostic methods, such as vaginal cytology, progesterone level monitoring, and luteinizing hormone (LH) surge detection, to precisely pinpoint ovulation [36]. Vaginal cytology provides valuable insights into the stage of estrus, while serial progesterone assays offer a reliable indicator of ovulation timing, as progesterone levels rise sharply prior to ovulation. Additionally, LH surge detection, though more challenging to measure, serves as a critical marker for predicting the optimal breeding window [37].

Despite the availability of these advanced techniques, many practitioners still rely on outdated methods or fail to integrate these tools effectively. This oversight can lead to suboptimal breeding outcomes, including reduced conception rates and increased risks of reproductive complications. Therefore, it is imperative for veterinarians and breeders alike to adopt a more evidence-based approach to estrus monitoring, leveraging the latest diagnostic tools and techniques to ensure precise ovulation determination.

In conclusion, the accurate determination of ovulation remains the cornerstone of successful canine reproduction. By embracing modern monitoring methods and addressing the gaps in current practices, breeders and veterinarians can significantly enhance breeding outcomes and contribute to the overall health and sustainability of canine populations.

#### 2.5.1. Breeding Soundness Examination

The first step of estrus monitoring is the breeding soundness examination (BSE). This BSE is aimed at identifying animals (females and males) that could have challenges with fertility at the time of the examination [33]. It is divided into two parts: a general examination and a specific examination of the reproductive system. The objective of the first part of BSE is to ensure that the bitch (or the stud dog) is able to mate and is healthy and able to achieve a normal pregnancy. The general examination consists of the following:

○ Observation for symptoms of endocrinopathy (for example, dermatological issues) that may indicate hormonal imbalances;
○ Examination of the oral cavity and teeth, as good oral health is essential; insufficient oral hygiene may lead to neonatal infections due to maternal licking;
○ Evaluation of the mammary glands to rule out anatomical abnormalities or the presence of tumors that could impair lactation;
○ Exclusion of musculoskeletal disorders, which could cause pain during mating (leading to unsuccessful reproduction) or worsen due to pregnancy.

If endocrinopathy is suspected, additional diagnostic tests are required to confirm or rule out the condition. Some endocrinopathies can affect fertility, while others may be heritable disorders, making it advisable to exclude the affected females from the breeding program [33]. Early detection of hereditary diseases is crucial for improving population health. For instance, canine atopic dermatitis is an immunological disorder influenced by both genetic and environmental factors. The heritability of this condition has been demonstrated in dogs and humans [38,39,40]. Therefore, implementing selection criteria to reduce the prevalence of atopic dermatitis would be advisable, particularly in breeds predisposed to developing the disease.

The second part of the BSE is the specific genital examination. This part has different objectives:

○ Evaluation of the anatomy of the vagina to ensure that mating and natural whelping are possible;
○ Evaluation of the vaginal health;
○ Evaluation of the uterus and ovaries to exclude diseases that could impair fertility;
○ Estimation of the ovulation date using progesterone assays and ovarian ultrasound.

#### 2.5.2. Evaluation of the Vagina

Regarding the evaluation of the vagina, two essential examinations are recommended: vaginal palpation and a vaginal cytology, with the latter ideally performed at least once per estrous cycle. On the day of the vaginal examination, the vaginal swab for vaginal cytology should be taken before palpation to avoid contamination of the vagina by the gel used for palpation or by bacteria introduced by the operator.

The vaginal palpation is essential in detecting vaginal anatomical abnormalities and assessing the female dog’s tolerance to examination. If the bitch is intolerant to vaginal palpation, it does not mean that she will not tolerate natural mating in all cases. However, it is a good test for the owner to anticipate if an insemination might be necessary and to prepare for this option. For example, in the case of a vaginal palpation intolerance it could be advised to try natural matings in the morning, allowing for time to arrange an artificial insemination in the afternoon if required. Additionally, owners should verify in advance whether a veterinarian near the mating location is available to perform the procedure on the appropriate day.

Among congenital vaginal malformations, the most commonly encountered anomalies include vestibulovaginal stenosis and vaginal septum.

**Figure 1:**
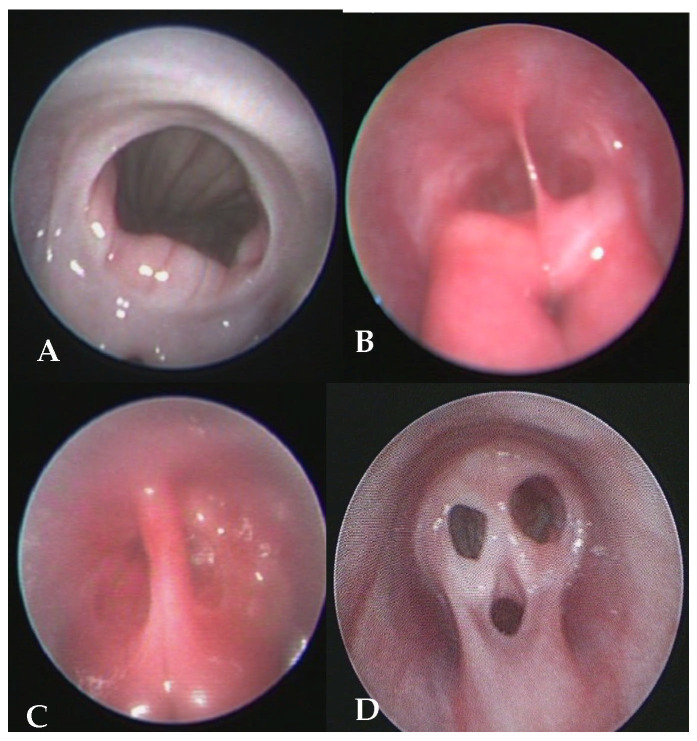
Endoscopic view of different vaginal malformation; (**A**): vestibulovaginal stenosis; (**B**): thin vaginal septum that was cut with the finger; (**C**): thick vaginal septum; (**D**): thick and 5 cm-long vaginal septum.

**Figure 2:**
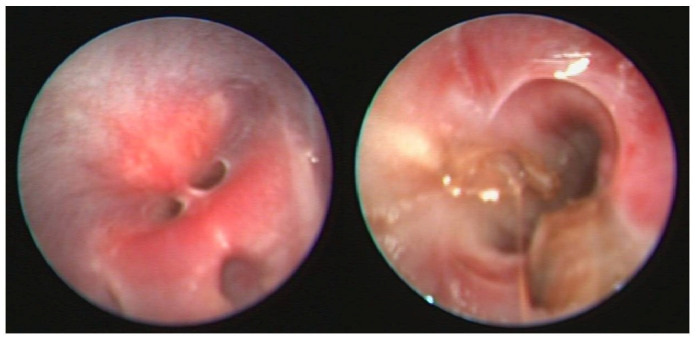
Endoscopic view of a vaginal septum on the (**left**) and after its removal by electrosection on the (**right**).

Vestibulovaginal stenosis often results from a persistent hymen, leading to a narrowed vestibulovaginal junction. Vaginal septa, on the other hand, can vary in presentation, ranging from thin ligamentous bands that can be manually disrupted to complete septa that divide the vaginal canal into two separate lumens. These malformations arise due to incomplete fusion of the paramesonephrotic ducts and could contribute to mating failure and, in some cases, dystocia [41]. Early diagnosis of such abnormalities within the estrus cycle allows for potential endoscopic removal of the septum, improving reproductive outcomes. If diagnosed later, or if the risk of dystocia is deemed high, elective cesarean section may be recommended to prevent complications during parturition. Figure 1 illustrates various types of vestibulovaginal malformations, while Figure 2 demonstrates the electrosection removal of a vaginal septum.

Figure 1 shows various types of vestibulovaginal malformations, while Figure 2 illustrates the electrosection removal of a vaginal septum.

Ensuring vaginal health before mating is essential. Monitoring the reproductive cycle with vaginal cytology at the beginning of the cycle is highly recommended as it allows for tracking the evolution of vaginal epithelial cells [42,43,44,45,46,47]. This method helps to determine the optimal timing for progesterone assays, which should be initiated when the transition from proestrus to estrus is observed. The combination of vaginal cytology and progesterone testing has been shown to improve the accuracy of estrus determination and allows for a more precise determination of the optimal mating window. This combined approach can reduce the frequency of unnecessary hormone testing, providing a more cost-effective and practical strategy for clinical veterinarians involved in canine reproduction [37,43,44]. However, some breeders may view this examination as an unnecessary financial burden. If the breeder does not wish to follow the estrus cycle with this technique, at least one vaginal smear during the estrus cycle is advisable to assess vaginal health.

Recent studies have investigated the impact of vaginal bacterial proliferation on fertility in the bitch [48,49].

Groppetti et al. (2012) [48] examined vaginal swabs from 45 bitches on the fifth day of proestrus. Bacteria were observed in all samples, with 79.1% yielding positive cultures, and 76.5% in pure culture. The most frequently isolated bacterial species in pure culture were *Enterococcus faecalis*, *Streptococcus haemolyticus*, *Pasteurella multocida*, *E. coli*, *Klebsiella pneumoniae*, *Proteus mirabilis*, *E. coli haemolyticus*, *Arcanobacterium pyogenes*, *Streptococcus* spp., *Staphylococcus* spp., and *Acinetobacter* spp. Moreover, 83.7% of vaginal smears showed the presence of neutrophiles, which means that not all of them had a positive culture. Interestingly, no statistical significant correlation was found between the presence of neutrophiles (with or without bacterial phagocytosis) and bacteriological outcomes as well as pregnancy rates, challenging the assumption that bacterial phagocytosis is pathognomonic for vaginal infection and requires systematic antibiotic treatment [48].

A large-scale study by Leps et al. (2024) [49] analyzed vaginal cultures from more than 23,000 bitches. Unlike Groppetti et al. [47], they predominantly observed mixed cultures, with the most common bacterial species being *Escherichia coli*, beta-hemolytic Streptococci, coagulase-positive Staphylococci, Pasteurelles, and aerobic sporulators, as well as *Streptococcus* spp. [49].

Given the increasing restriction on antibiotic use, antibiotic treatment should be reserved for bitches whose fertility might be impaired by a genital tract infection. Vaginal culture should be systematically performed before initiating antibiotic therapy, and concurrent vaginal cytology should be conducted while awaiting culture results. Antibiotics should be prescribed not solely based on a positive culture but rather in cases where a genital infection is confirmed by cytology and/or there is a history of infertility associated with genital tract infections [48].

#### 2.5.3. Progesterone Assays and Ovarian Ultrasound

Progesterone assays are widely recognized as the gold standard for precisely determining the timing of ovulation. Given their extensive documentation in numerous studies and reviews, their methodological details will not be reiterated here [35,37,50,51,52].

Instead, this review aims to highlight key analytical considerations that may influence the interpretation of the progesterone values and, consequently, the accurate determination of the ovulation timing. Notably, comparative studies on different assay methods consistently indicate that, while most techniques demonstrate strong reliability, they yield varying results for the same blood sample. Thus, the critical factor in utilizing progesterone assays is not merely the analytical device itself but rather a thorough understanding of its reference values and, more importantly, the dynamic changes in progesterone levels throughout the estrous cycle.

Additionally, it is not uncommon for two instruments from the same manufacturer, employing the same assay methodology, to produce discrepant progesterone values for an identical blood sample. Table 1 gives an overview of the different threshold values relevant to key phases of estrus monitoring, as reported in the literature and laboratory data [51,53,54,55,56].

Proper blood handling conditions are crucial, as they can significantly impact assay results [57]. The following are some widely accepted recommendations:

○ The same kind of tubes should be used consistently throughout the whole estrus cycle monitoring (either plasma or serum, according to the laboratory recommendations). If both options are available, the same medium should be used for the entire cycle. Tubes containing gel separators should be avoided [58];
○ Lipemic plasma/serum should not be used [56];
○ Blood samples should be collected at the same time (either consistently in the morning or the afternoon) to minimize variability [58].

More practically, the key parameter to monitor ovulation is the progression of progesterone levels over time. If progesterone reaches the typical ovulation range for the assay in use, ovulation should be confirmed by obtaining a follow-up blood sample 24 h later. A rapid rise in progesterone levels, exceeding the ovulation threshold the following day, serves as confirmation of ovulation. In specific cases, such as when using frozen semen, one study demonstrated that the pre-ovulatory progesterone slope plays a critical role in whelping success. A steeper progesterone increase prior to ovulation has been associated with improved reproductive outcomes [51].

The use of ultrasound to determine the time of ovulation has gained considerable importance in both clinical practice and research. This advanced imaging technique is an indispensable diagnostic tool that provides a comprehensive set of data on ovarian physiology. In particular, ultrasound facilitates the detailed monitoring of follicular growth, enables the precise detection of ovulation, and allows for the subsequent formation of the corpus luteum to be observed. In addition, it plays a crucial role in confirming blood progesterone concentrations, especially in cases where the progesterone testing system has been modified in the clinical setting, necessitating the validation of new reference ranges [52,59,60,61,62,63,64]. Beyond its diagnostic utility, ultrasonography provides valuable insights into ovarian activity that can be used to assess fertility potential. By quantifying the number of follicles present in the ovaries—or at least by assessing their relative number—the likelihood of high or low fertility can be inferred. While this approach does not allow for accurate prediction of embryo yield, as variables such as fertilization efficiency need to be taken into account, it does provide important prognostic information on reproductive capacity. For example, the absence of ovarian activity or the detection of only a limited number of follicles may suggest a reduced probability of a large litter, thereby providing valuable insights into the expected litter size. However, these assessments have not been statistically verified in the literature.

Furthermore, in specific clinical contexts, e.g., when anovulatory cycles are suspected, the integration of ultrasound findings with vaginal cytology and serial progesterone measurements can provide particularly informative results. This multimodal diagnostic approach increases the accuracy of reproductive assessment and contributes to a more nuanced understanding of ovarian function. In summary, ultrasonography is a versatile and essential tool in reproductive medicine, providing both diagnostic accuracy and prognostic value in the assessment of ovarian dynamics and fertility [62].

### 2.6. Mating and Insemination Management

Mating is a decisive factor for successful reproduction. Morever, a bitch can not be qualified as unfertile if proper matings have not been observed. Rather than the frequency of mating, the exact timing of insemination plays a crucial role in reproductive success, especially given the unique reproductive physiology of the bitch [65].

A characteristic feature of canine reproduction is the delayed maturation of the oocytes after ovulation, which differs significantly from that of other animal species. At the time of ovulation, the oocytes are still immature and require additional time to reach metaphase II, during which fertilization can take place. Studies have shown that resumption of meiosis occurs hours after ovulation, with oocytes being capable of fertilization between 48 and 83 h thereafter [65,66]. The optimal time for mating or artificial insemination is, therefore, based on these physiological findings. Although fertilization up to eight days after ovulation by transcervical insemination (which was performed surgically in the cited study) has been documented in exceptional cases, best reproductive practices advise against insemination outside the estrus period. This recommendation is based on evidence suggesting that aging oocytes are associated with an increased risk of embryonic resorption, which ultimately reduces reproductive success [67].

If the male is readily available, mating once daily from the day after ovulation until the third day post-ovulation is a good time frame. If only a single mating is feasible, the second or third day following ovulation is the most favorable choice [65,66].

When using artificial insemination and, especially, transcervical insemination (TCI), the ideal timing for a single insemination is 2 to 3 days after ovulation. This timing has been supported by research, including the study by Thomassen et al. (2006) [68], which demonstrated improved outcomes when using frozen semen—the most challenging type of semen for successful insemination. Interestingly, their study found that whelping rates in bitches inseminated in this optimal time window did not differ significantly from those inseminated twice. However, litter size varied, highlighting the importance of precise timing in maximizing reproductive success [68].

The success of insemination depends primarily on two factors: the exact timing of the procedure and the quality and type of semen used. Both play a decisive role in determining fertility. Of course, male fertility also has a significant influence on the success of insemination or natural mating, and the male causes of infertility are discussed in detail in Section 4. Table 2 provides some examples of results found in the literature to compare the different options.

In short, the author’s advice for promoting fertility in relation to the timing of inseminations and taking into account the maturation of the oocytes, but also their survival time and the survival time of the sperm in the female genitalia, is as follows [66,68]:

○ Natural matings or intravaginal insemination: days 1 and 3 after ovulation;
○ Transcervical insemination with fresh semen: days 1 and 3 after ovulation if two inseminations are possible, or day 2 or 3 if only one is possible;
○ Transcervical insemination with chilled semen: days 2 and/or 3 after ovulation;
○ Transcervical insemination with frozen semen: days 3–3.5 after ovulation.

It should be noted that some clinicians opt for insemination on day 4 after ovulation, and successful results have also been reported with this approach. However, the protocol proposed here reflects the authors’ current clinical practice and is intended as a practical guide for general veterinary use. Although alternative timings may be equally valid, particularly in specialized facilities, the method described has consistently produced satisfactory results under routine clinical conditions.

Although surgical insemination is still frequently cited in the literature, a growing number of countries are moving away from this practice on ethical grounds and have already imposed restrictions on its use, particularly in light of the increasing availability and success of transcervical insemination (TCI). This shift raises the important question: Are the results of TCI comparable to or better than those of surgical insemination? Although few studies have directly compared the two techniques, the available data suggests that TCI results in pregnancy rates and litter sizes that are at least equivalent, if not superior, to those of surgical insemination [69,71,72].

Interestingly, studies have shown that TCI can increase pregnancy rates by more than 30% compared to intravaginal insemination [73,74]. This could be due to several reasons. Firstly, poor management of the insemination technique is suspected. In TCI with the endoscopic technique, the deposition of semen in the uterus is safe because the veterinarian can see the catheter being passed through the cervix. If the cervix cannot be crossed, the semen is deposited in the cranial part of the vagina near the entrance to the cervix. With vaginal insemination, the catheter may be accidentally inserted into the urethra or may not reach the optimal depth in the vagina. Additionally, the sperm must cross the cervical barrier, which can lead to a significant reduction in sperm quantity and viability, reducing fertility potential. Furthermore, the vaginal environment itself can affect semen quality, further worsening the outcome [74].

If matings or vaginal inseminations (especially if performed by the breeder without semen quality control) have been performed at the optimal time during the bitch’s fertile period and still do not result in pregnancy (excluding post-conception causes of infertility), the author advises to consider a TCI as the next step. Practically, this approach not only allows for the semen quality to be checked on the day of semen use, but also ensures that the cervical barrier is bypassed, maximizing the effectiveness of the entire ejaculate. If this approach also fails, a thorough investigation of possible female—and male—specific causes of infertility should be carried out, as described in the following sections.

### 2.7. Impact of Stress on Fertility

To conclude this section, it is important to briefly discuss the possible effects of stress on fertility. Although specific studies on the role of stress in canine reproduction remain limited, studies in other animal species—particularly humans, rodents, pigs, and cattle—have clearly shown that stress has a significant impact on reproductive success [75,76,77].

Stress can affect various reproductive processes, including sexual behavior, the hypothalamic–pituitary–gonadal axis, ovulation, fertilization, early embryonic development, fetal growth, and even maternal care. Depending on the type of stress, it can act through endocrine, neuroendocrine, or metabolic pathways and ultimately affect reproductive outcomes.

Different types of stress should be considered when investigating infertility in clinical cases:

- Psychological stress, e.g., related to housing conditions (e.g., confinement, lack of environmental enrichment), social hierarchy in the group, or the relationship between owner and animal;
- Environmental stress, including thermal stress (particularly heat stress) and suboptimal nutrition;
- Metabolic or physiological stress that may be caused by underlying systemic disease or chronic inflammation.

While further research is needed to clarify these mechanisms specifically in dogs, knowledge of these factors may be beneficial in clinical decision-making, particularly in cases of unexplained infertility.

## 3. Female Preconceptional Causes of Infertility

After rulling out all breeding causes for infertility, it is important to search for a specific problem in the bitch or stud dog. The male will be discussed in the next part (Section 4).

The reproductive status of the bitch can be assessed through various methods. A common question posed during the breeding soundness examination (BSE) is whether the animal exhibits regular estrous cycles. To provide clarity, we have structured this section based on the response to this question, categorizing bitches into those with regular and irregular cycles. In each section, the specific causes are described, and the most important points for their treatment are discussed.

### 3.1. Bitches with Irregular Cycles

The duration between two estrus cycles can vary in different bitches. It is important that this interval remains stable in one and the same animal, for example, every 6 months, every 8 months, or every 12 months. If this interval is maintained—excluding very short cycles (under 4 months), which may also indicate a health issue—the bitch is considered regular. Otherwise, she is classified as irregular [78].

If the bitch is irregular, two primary categories of causes should be investigated: ovarian dysfunction and endocrine causes of infertility.

#### 3.1.1. Ovarian Causes

In young bitches, usually in their first heat cycle, a so-called “split heat” is sometimes observed. Some breeders also speak of “false heat”, which can be defined as an abnormally short duration of proestrus or estrus, accompanied by an abnormal increase in serum progesterone. A new cycle begins a few days to weeks later and is usually normal [78,79].

Another cause of a shortened interval without underlying genital disease is the anovulatory cycle. The bitch begins the estrus cycle, progesterone increases, but never reaches the ovulation rate until the vaginal smear typical of estrus (majority of keratinized cells) finally indicates the end of estrus. This affects about 1% of estrus cycles and shortens the inter-estrus interval, but has no effect on fertility in the next estrus cycle. In fact, a new cycle, which is normally normal and fertile, begins in the days or weeks following this atypical phenomenon [8,79].

Various causes have been suggested to explain the anovulatory cycle: a central defect in the production of an efficient LH surge due to insufficient production of GnRH by the hypothalamus or a peripheral origin due to a disturbance in ovarian estrogen production that results in no LH surge being triggered or the ovaries not responding to a normal LH peak [79].

An irregular cycle can also be artificially suspected if the signs of estrus occur only rarely or not at all. This is commonly referred to as “silent oestrus”. The bitch may have a normal cycle with no signs of estrus (no discharge from the vulva or swelling, the bitch may also not attract males), so the owner assumes a prolonged anoestrus [8]. If a silent heat is suspected, it is advisable to either check the progesterone concentration in the blood every two weeks or to take a vaginal smear once a week to detect the first signs of keratinization of the vaginal cells and the start of the oestrus cycle. If two vaginal swabs with two progesterone tests are taken too far apart, there is a risk of missing the start of the cycle and not recognizing estrus until after the fertile period.

Luteal insufficiency also needs to be addressed. However, if it is a cause of shortened inter-estrus interval, it is a post-conceptional cause of pregnancy failure.

Persistent estrus can be defined as estrus (confirmed by repeated keratinized vaginal smear) lasting longer than 21 days [79]. However, if an idiopathic cause is suspected (all other causes have already been ruled out), bitches can be monitored closely for a prolonged period as long as they show no signs of bone marrow aplasia or uterine disease. If the estrus cycle is abnormally long, mating should not be attempted. Treatment of idiopathic estrus with human chorionic gonadotropin (hCG) has been described but is often disappointing. It can lead to negative consequences, such as pyometra, and does not prevent recurrence [79,80]. As a result, the authors recommend ovariohysterectomy in cases that would take too long.

There are two other main causes of prolonged estrus in the bitch: the presence of ovarian cysts or tumors.

Ovarian cysts are not uncommon in the bitch and can account for up to 80% of ovarian disease [81]. However, it is difficult to determine their nature as only histology can give an answer if they do not produce hormones. Ovarian cysts can be unilateral or bilateral and can develop as a single entity or as a polycystic disease. The different possible histologic forms are follicular cysts, corpus luteum cysts, cystic corpus luteum cysts, rete ovarii cysts, and cysts of the subcutaneous epithelium. According to various studies, the latter two are the main form of ovarian cysts in the bitch, and their effects on fertility are not known [82,83,84]. Follicular cysts are the form responsible for persistent oestrus. They can be solitary or multiple and vary in size. Caution should also be exercised when using deslorelin implants. One case has been described in the literature in which a follicular cyst developed after implantation, and the author has observed some cases that spontaneously regressed after removal of the implant (personal data, unpublished) [85]. The treatment of ovarian cysts was described in a case series, in which the type of cyst was not specified [86]. In this article, repeated treatment with hCG (between 450 and 3000 IU per animal) and/or buserelin (GnRH analog between 0.8 and 6 mg per animal) was attempted, with variable results depending on the number of treatment repetitions, although the success rate never exceeded 66.7% [83,86]. Ultrasound-guided aspiration of cyst fluid or surgical removal of cysts has also been described in cases of single cysts or few well-observed cysts, although recurrence is possible. In cases where treatment fails, ovariohysterectomy is the definitive treatment [83].

Ovarian tumors account for approximately 1% of tumors in dogs [82]. They can be classified as epithelial tumors (papillary adenoma or adenocarcinoma, cystadenoma), sex-cord stromal tumors (including granulosa cell tumor GCT, thecoma, and interstitial and Sertoli cell tumors), germ cell tumors (dysgerminoma, teratoma, and embryonal carcinoma), or other types [87]. The tumor that could be responsible for persistent estrus is, in most cases, the GCT. Other tumor types can lead to variable cycle changes, such as irregular heat, prolonged anoestrus, or primary anoestrus, especially in the case of teratoma, a tumor that develops in younger bitches. Uterine changes may be associated with ovarian tumors (e.g., pyometra or CEH) and abdominal pain or enlargement, but the absence of clinical signs is also possible. Epithelial tumors (adenocarcinoma and adenoma) and CGT are the most common tumor types, with the former type accounting for 40–50% of ovarian tumors and CGT accounting for about 50% (often referred to as the most common ovarian tumor by the authors). Epithelial tumors are more often bilateral than other tumors, and the presence of metastases is reported to be about 48%. CGTs tend to be unilateral; their appearance can be very cystic and can be mistaken for a polycystic ovary (and vice versa). Metastases of GCT occur in about 20% of cases [82].

Ovarian tumors are easy to detect during an ultrasound examination. However, their nature cannot yet be determined by any imaging technique, and histologic analysis is required [88]. However, abdominal ultrasound allows for the detection of abdominal effusion or signs of local or distant metastasis to other abdominal organs and is essential for the evaluation of ovarian neoplasms. Diagnosis and treatment are performed simultaneously with ovariohysterectomy [89].

The prognosis of malignant ovarian tumors in the bitch is still considered good if aggressive treatment (ovariohysterectomy and adjuvant treatment in certain cases) is performed as early as possible. In a recent case series of 18 bitches, CGT was the most common form of ovarian neoplasia, followed by dysgerminoma and adenocarcinoma. In this group, the overall median survival time was 1004 days, with the median survival time of bitches with metastatic disease at diagnosis being approximately one year (391 days). Estrus symptoms were only observed in dogs with CGT, and metastatic disease was not observed in dogs with CGT, only in two dogs with dysgerminoma and two with adenocarcinoma. In this study, the median survival time was negatively associated with tumors invading more than one ovary, the presence of metastatic disease, and invasion of the lymphatic space [89].

**Figure 3:**
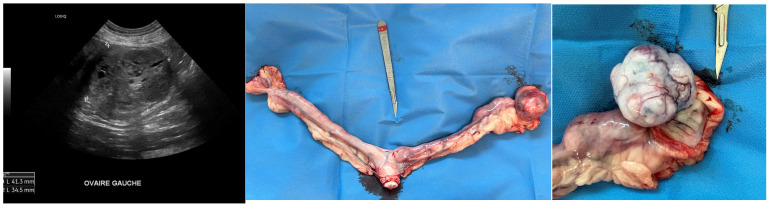
Example of CGT in an English Bulldog bitch presented for persistent signs of estrus. The (**left**) image is the ultrasound image of the mass; in the (**middle**), the internal genitalia after ovariohysterectomy, and on the (**right**), an image of the left ovary from the ovarian bursa. The scale is given by the surgical blade size 11.

Figure 3 shows an example of unilateral CGT in a bitch that was presented at the referral clinic for persistent estrus (vulvar swelling and discharge for more than 4 weeks).

Finally, in irregular cycles or primary anestruses, especially in young bitches or if an abnormal genitalia is observed, a disorder of sexual development should also be suspected [90].

#### 3.1.2. Endocrine Causes

First of all, when looking for an endocrine cause of infertility, it is always important to exclude a possible exogenous treatment that could affect the cycle or the health of the uterus. The effect of exogenous addition of progestogens has been discussed previously. In bitches showing signs of persistent estrus, it is always interesting, especially if no signs of ovarian disease are observed, to rule out treatment by the owner that could be transmitted to the dog [91].

Although various endocrine disorders are often cited as possible causes of irregular oestrous cycles, hypothyroidism is the only one currently thought to have a direct effect on cycle regularity.

Hypothyroidism is suspected of causing cycle abnormalities such as prolonged anoestrus, silent oestrus, or increased cycle frequency. The influence of thyroid hormones on the sexual hormone axes could be related to the production of prolactin. TRH (thyroid-releasing hormone) is elevated in affected bitches. TRH may interfere with dopamine, which normally modulates prolactin production. An elevated TRH level can then cause hyperprolactinemia and reproductive disorders [92,93,94]. In recent case series, however, no influence on the estrus cycle in hypothyroid bitches has been found [95,96]. In the author’s experience, the effect of hypothyroidism on the reproductive cycle is rare, but in at least one case, a healthy litter was diagnosed after treatment. Although the impact of this condition on fertility is still controversial, it should be investigated in cases of irregular cycles or unexplained infertility.

Hyperthryoidism is uncommon in the bitches. However, one case has been published in which a bitch was presented for prolonged oestrus. After a normal general and genital examination, hormonal investigations were carried out, which revealed hyperthyroidism. Further investigations revealed that it was a case of nutritional hyperthyroidism associated with a BARF diet (bones and raw food). The diet contained meat from the head and neck area of cattle, which inadvertently contained thyroid tissue and led to an exogenous uptake of thyroid hormones. After a change in diet, thyroid function returned to normal and the bitch subsequently gave birth to a healthy litter [97].

Hypercorticism was also cited as an explanation for the bitch’s infertility. Elevated glucocorticoids over a prolonged period in the bitch would cause prolonged anoestrus in over 75% of bitches. Cushing’s syndrome often affects middle-aged to older dogs that are not engaged in reproduction. However, in the case of prolonged anoestrus, especially in younger bitches, exogenous treatment with corticosteroids should also be investigated (as a reminder, questions about recent or chronic treatments administered to the bitch should always be asked at the beginning of the consultation) [8].

### 3.2. Bitches with Regular Cycles

#### 3.2.1. Timing of Breeding and Idiopathic Infertility

As already mentioned, incorrect mating timing is still the main cause of infertility. Another occasionally reported but poorly understood factor is the apparent incompatibility between a particular male and a particular female, even though both are individually fertile. This phenomenon lacks a scientific explanation, yet breeders universally recognize its baffling reality. Despite investing substantial resources in infertility treatments to produce a litter from a carefully selected pair, efforts often fail—only for the female to later mate effortlessly with an unintended male. To make the hypothesis of incompatibility, all known infertility causes need to be excluded. When all other potential causes are ruled out, it remains critical to advise pairing the female with a different male before concluding she is infertile.

This type of infertility, perpetually suspected but never proven, stands as one of the most exasperating challenges in breeding.

#### 3.2.2. Uterine Environment

The uterus is the organ of pregnancy development. As such, uterine health is also a key to a successful mating. However, most of the uterine-related causes of infertility are better classified in post-conceptional causes of infertility and, so, outside of the scope of this manuscript, but they are nicely and deeply explained in a recent manuscript [6].

An anatomical defect of the uterus can obviously induce infertility. However, a large-scale study including 32,660 dogs published in 2010 showed that uterine congenital abnormalities are rare and concerned only 0.05% of the included dogs. Three abnormalities were observed: unicornuate uterus in 11 dogs, segmental agenesis of one horn in 3 dogs, and uterine horn hypoplasia in 1 dog. Interestingly, 50% of dogs with unilateral horn agenesis had also experienced ipsilateral kidney aplasia if their kidneys were evaluated [98]. These congenital abnormalities are rare but may have an impact on fertility, and an ultrasound examination of the infertile bitches should always be performed.

CEH is a major uterine disease that will impair fertility. This alteration has been described in Section 2.2 of this review and, as said earlier, is a post-conceptional cause of pregnancy failure. However, its diagnosis can be made before mating and may lead to a change in the breeding program of the breeder [13,14,15,18].

Another cause of infertility described is endometritis. Endometritis is considered more likely a post-conception cause of infertility. However, its treatment can be conducted before conception and should, therefore, be discussed at the time of estrus evaluation. In the bitch, various studies have been conducted to evaluate this alteration, whose only clinical sign in most cases is infertility. In a study including 26 infertile bitches, 26% had uterine cytology compatible with endometritis, and 70% of them had positive bacterial growth on uterine samples [99]. The causes of endometritis in the bitch are still poorly understood. The uterus may be a sperm reservoir, and the arrival of semen triggers an inflammatory response in the endometrium, which, in some cases, is overexpressed. This, possibly in conjunction with uterine clearance failure, would lead to endometritis [9,10]. The diagnosis is made clinically (the presence of intraluminal fluid in the uterine horns in diestrus on ultrasound images may also be a sign) and can also be made by transcervical or surgical cytology and biopsy [11,99,100,101]. In a recent review, this affection was compared in the main domestic species [102]. Various protocols have been proposed to treat endometritis and to create a good environment for the arrival of the embryo in the uterus, but there is no consensus on this topic. The use of antibiotics, especially in bitches with CEH, is often cited and is justified by the finding that many bitches with endometritis also had positive culture on uteirine cytology in the study described above [99]. The use of oxytocin to help uterine clearance has also been proposed (0.08 IU/kg injected after the second day of mating), although there is not yet sufficient data to draw a conclusion about its efficacy [10]. Finally, the authors also mentioned the use of non-steroidal anti-inflammatory drugs (NSAIDs), which still need to be evaluated [10].

Finally, it is also important to consider the time needed by the uterus to complete its involution and be ready to carry another litter. The duration of involution in the bitch is long, around 84 days, which corresponds to almost 3 months post-whelping [102]. In short-cycled bitches, for example bitches that have a new cycle every four months, it is recommended to always wait at least one or two cycles to allow the uterus to rest. If a cycle needs to be medically induced, it is recommended to wait at least 5 months after the last onset of heat [102].

#### 3.2.3. Infectious Diseases

As already described, the uterus is attacked by ascending pathogens during the estrus period. Health of the vagina is, therefore, important to ensure the global health of the genital tract. However, it is also known that cultures performed on a vaginal sample are not conclusive as to the contents of the uterus, especially at the onset of diestrus. The consequence of this is that if a pathogen is suspected, it should be looked for where the problem occurs, namely, in the uterus and not in the vagina. Moreover, a positive result should be carefully interpreted in conjunction with the presence of clinical signs and other complementary examinations (e.g., signs of inflammation in the cytological analysis, leukocytosis, or vaginal discharge).

Although the microbiota of the canine uterus remains poorly characterized, recent evidence suggests that the canine uterus is not a sterile environment, even in healthy and fertile individuals. In a recent study, bacteria such as *Bacillus* spp. and *Pseudomonas* spp. were detected in the uterus of clinically normal, fertile bitches, challenging the long-held assumption of uterine sterility [103]. In other animal species, the role of the uterine microbiota in reproductive function has been studied in more detail. In mares, for example, an imbalance in the endometrial microbiota (dysbiosis) has been associated with the development of subclinical or chronic endometritis, a recognized cause of infertility. In women, changes in the composition of the uterine and vaginal microbiota have also been associated with implantation failure, chronic endometritis, and poor outcomes in assisted reproductive technologies [104,105,106,107,108,109]. These findings highlight the increasing importance of the reproductive tract microbiome as a potential factor in fertility, even in the absence of overt infection. Although further research is needed to elucidate these mechanisms in dogs, the presence and potential impact of uterine microbiota should be considered in the assessment of unexplained infertility.

However, only a limited number of infectious agents have been clearly shown to adversely affect fertility, and in some cases, this has only been confirmed under experimental conditions. These include *Brucella* spp., *Mycoplasma canis*, Herpesvirus, Parvovirus, *Toxoplasma gondii*, and *Neospora caninum*. Moreover, these are more likely to be responsible for post-conceptional loss and not for preconceptional loss [110,111]. It is essential to exclude a pathogen when infertility is observed in a bitch or a stud dog, because as a zoonotic agent, it has implications for human health: *Brucella* spp. can cause brucellosis. Recently, cases of brucellosis affecting humans have been published, and this possibility should never be neglected [112,113].

## 4. Male Preconceptional Cases of Infertility

When addressing preconceptional causes of canine infertility, a comprehensive discussion of male fertility is indispensable. Male reproductive health directly influences breeding success rates, as any impairment in the male’s fertility can compromise the viability of conception. Notably, one study posits that male-related factors account for 40–50% of pregnancy failures in dogs [114]. While this topic has been extensively reviewed in the literature [114,115,116,117,118], this section will focus on clinically relevant aspects of male infertility that can be readily assessed in general veterinary practice. In particular, we will look at the performance of breeding soundness examination (BSE), common mating disorders, and assessment of semen quality, with an emphasis on underlying causes and practical management where possible.

The BSE is as important for males as it is for females and serves as a basic diagnostic step. A male dog that is unable to mate naturally or unwilling to copulate must either be artificially inseminated or excluded from the breeding program if semen collection is not possible. The BSE should follow a systematic approach [114]:

1. General physical examination: assessment of general health to detect systemic diseases (e.g., endocrine disorders such as hypothyroidism) or hereditary diseases that could affect semen quality or the health of the offspring;
2. Genital examination and semen analysis: assessment of reproductive anatomy (e.g., testicular size, penile structure) and performance of a semen analysis (volume, concentration, motility, morphology) to confirm functional fertility.

This structured examination ensures that preconceptional male infertility is accurately diagnosed and allows for informed decisions to be made for breeding management. Clinicians must prioritize the assessment of male fertility alongside the assessment of females to prevent avoidable reproductive failure.

### 4.1. Mating Disorders

#### 4.1.1. Anatomical Defects

Certain anatomical anomalies can lead to unsuccessful or impossible mating. A thorough examination of the male, particularly in comparison with the female, is essential—especially in breeds with pronounced sexual dimorphism in terms of skeletal structure and body structure—to identify significant size differences that could hinder mating [114].

**Figure 4:**
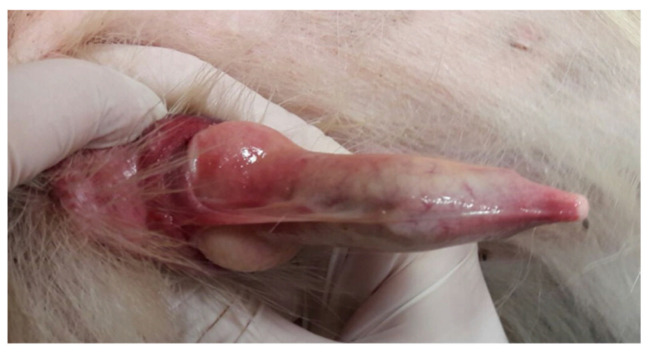
Persistent penile frenulum in a young dog presented for frenetically licking the prepuce.

Figure 4 shows an example of such an anatomical abnormality: a persistent penile frenulum. This condition can prevent full penile thrusting, cause penile deviation during erection, or lead to pain that interferes with mating behavior. In addition, excessive hair in the area of the frenulum can hinder proper protrusion of the penis, possibly leading to mismating.

A thorough genital examination should confirm that the penis moves freely within the prepuce and can be easily extruded and retracted, thereby ruling out conditions such as phimosis or paraphimosis. Additionally, both testicles should be normally developed, non-painful, and present within the scrotum [114].

Genetic disorders can also lead to semen disorders and infertility [90].

#### 4.1.2. Pain and Behavior Disorders

Any pain that could be felt during mating can lead to a mating failure. The pain can be located in the genitalia or any part of the body that is used during mating behavior. For example, dogs with hip dysplasia or cruciate ligament defects might be reluctant to mate. Palpation of the penis during the BSE can reveal a pain that could be linked to a previous trauma of the os penis. Inflammation and/or infection of the prepuce or the penis (balanitis, posthitis or both) but also the prostate gland (prostatitis) or the urinary tract (urethritis or cystitis) can result in pain during erection and/or ejaculation [114,119].

Behavior disorders should also be considered. It is not rare to see dogs reluctant to mate in the presence of the owner or other humans. If the dog has been previously disrupted from mating for any reason, he might associate the willingness to mate with a bad behavior and can be reluctant to mate when it is finally desired. The presence of other males can also lead to a decrease in libido or aggressiveness from the female toward the male. If an environmental condition is suspected to lower the male’s libido, changes should be made. However, semen and artificial insemination can also be a solution, although dogs can also be reluctant to this manipulation [119].

A lack of libido can therefore be psychological, idiopathic, or hormonal. Androgen insufficiency associated with a lack of libido is a rather rare condition, but should also be investigated [119]. The assessment of the hormonal state is discussed later in this article.

#### 4.1.3. Ejaculatory Disorders

Ejaculatory disorders can be difficult to assess, especially in dogs that are only used for natural breeding and have a normal libido and willingness to mate. One possibility to check if the dog has ejaculated is to perform a vaginal swab within 24 h of mating. However, this technique is not 100% diagnostic, and smears where sperm are observed are also not certain of sperm viability [119].

Retrograde ejaculation is rare but possible in dogs. It can be the cause of oligozoospermia (due to an incomplete ejaculation, where a portion passes into the bladder) or azoospermia/aspermia. The diagnosis is based on a urine collection after semen collection and the detection of large amounts of sperm in the bladder. The causes of retrograde ejaculation in dogs are not exactly known, but a disorder of the bladder neck closure associated with neurological disorders (sympathetic blockade) is suspected. Treatment with phenylpropanolamine (proposed dosage: 4mg/kg orally once daily for 5 days before collection) and pseudoephedrine (4 to 5 mg/kg orally three times daily or 1 and 3 h prior to collection or breeding) has been shown to be helpful in reducing the number of spermatozoa in the bladder after ejaculation [114,120]. From the authors’ experience, the use of furosemide one or two hours before collection may also be useful (personal data).

If azoospermia is detected (i.e., the absence of spermatozoa in the semen), it must first be determined whether the dog has ejaculated or not, and a distinction must be made between obstructive and non-obstructive azoospermia. Obstructive azoospermia (or oligozoospermia) can be caused by a blockage of the second fraction of ejaculation. Several case reports of sperm granulomas and spermatocele have been published, two of which were the results of a surgical procedure (uretrostomy and vasectomy), and the other was probably caused by disruption of the hematotesticular barrier, causing an immunologic reaction [121,122,123,124]. Orchitis and epididymitis can also lead to this condition, and ultrasonography should always be performed to observe the aspect of these organs.

Non-obstructive azoospermia is caused by a defect in spermatogenesis, leading to an absence of sperm in the ejaculate despite no physical blockage in the reproductive tract [119].

To differentiate between obstructive and non-obstructive azoospermia, the level of alkaline phosphatase in the sperm is commonly used. This enzyme is produced by the epididymis and reflects the presence of the second fraction of the ejaculate. Studies have reported a cut-off value of 5000 IU/L, below which the ejaculate is considered incomplete, meaning that the azoospermia is obstructive or the dog has not ejaculated [115,125,126]. In this last case, the aspermia can be due to a problem with the collection procedure (the dog can have a lack of libido or needs to be trained to collect manually). In this case, the alkaline phosphatase in the semen is low because the dog is retaining semen (and can also have aspermia), but it is not a production problem. If this is suspected, the collection should be repeated under different environmental conditions with a bitch in heat that is friendly to the stud dog [114].

### 4.2. Semen Defects

In this part, we will only focus on the causes and diagnosis of impaired semen quality. It is important to note that there are only a few papers studying the relationship between the usual semen parameters included in classical semen analysis (concentration, mobility, and percentage of morphologically normal sperm cells) and fertility. However, the results of breeding are shown to be better when [23,68,127] the following factors are observed:

- More than 65% of the ejaculate’s sperm cells are progressive;
- More than 200 million progressive motile sperm are inseminated;
- The number of sperm cells in the ejaculate is high (when the quality of the ejaculate is moderate, it could be compensated by the quantity);
- The percentage of morphologically normal sperm cells inseminated is over 60–70%.

Moreover, the authors agree that when semen of lower quality is used, fertility can be improved by increasing the frequency of intrauterine artificial inseminations [69].

#### 4.2.1. Testicular, Epididymal, and Prostatic Causes

Alterations of the testes and prostate gland may cause a reduction in semen quality, impairing male fertility.

Different publications have shown that prostatic diseases, even in the early stages such as benign prostatic hyperplasia (BPH), but also more severe diseases such as prostatitis, can affect semen quality: increased DNA fragmentation and primary abnormalities, decreased mobility, and changes in the composition of seminal fluid [128,129,130,131,132].

One of the first signs of BPH is the presence of blood in the semen (hematospermia) that can sometimes be observed without any other clinical signs and is detected at the time of collection. Hematospermia associated or not with a lower semen quality should lead to the suspicion of prostatic syndrome and the conduct of complementary exams. Prostatic syndrome can be assessed by measuring the concentration of CPSE (canine prostatic-specific esterase), which reflects prostate enlargement and shows changes even in the early stages of benign prostatic hyperplasia (BPH), with a diagnostic threshold set at 52.3 ng/mL. However, when interpreting CPSE levels, caution is advised, as recent ejaculation can alter the values and lead to misinterpretation [133,134]. To go further, an ultrasound examination is mandatory. Formulas are available to mesure the prostatic volume (Prostatic volume (cm^3^) = length × width × height × 0.523) to compare with the expected normal volume taking into account the age and size of the dog (expected volume (cm^3^) = (0.867 × BW) + (1.885 × A) + 15.88) [135]. The global shape of the prostate and the presence of cavities (abscess or cysts) can be evaluated by ultrasound. Changes in the echogenicity of the prostatic parenchyma may be indicative of abnormal conditions such as acute or chronic prostatitis, or potentially prostatic neoplasia. Many articles and reviews have described the prostatic ultrasound evaluation (using bidimensional ultrasound, but also Doppler mode to evaluate the prostatic vascularization and other more recent techniques) and are not detailed here [63,135,136].

Different treatments have been proposed to treat the early stages of BPH. For example, the use of finasteride (an 5α-reductase inhibitor, 1.25 mg/dog once per day for 195 days with a return to pre-treatment volume after around 2 months when the treatment is ceased) or osaterone acetate (a progestogen/antiandrogen, 0.2–0.5 mg/kg/day for 7 days allowing for a prostatic regression within 7 to 28 days with a return to pre-treatment volume after around 5 months) are commonly used with success on stud dogs in which fertility need to be conserved. The use of osaterone acetate has been shown to preserve the fertility of the treated dogs, and this drug is registered for dogs. However, treatment may lead to a reduced volume of the ejaculate, accompanied by a temporary rise in the incidence of sperm tail abnormalities [132,137].

All diseases of the testis can lead to reduced semen quality. Different modifications can be observed: testicular neoplasia that can be easily detected on ultrasound examination, orchitis (acute or chronic and associated or not with epididymitis and prostatitis), and testicular atrophy (which can be linked to the age of the dog or the final consequences of other testicular problems). Orchitis and epididymitis can be infectious or autoimmune. Concerning the diagnosis, in the acute phase, one or both testes can be enlarged and painful, the scrotum can be swollen, and this can lead to general symptoms like lethargy, dysorexia, fever, and hind-limb lameness. In the chronic phase, sometimes no clinical symptoms are present except for testis dysmetria caused by the affected testis’s atrophy or fibrosis. Diagnosis is performed by ultrasound examination of the testes, cytology, and culture of the ejaculate [114]. Cytology by fine needle aspiration and ultrasound-guided biopsy of the testes has been described. Although authors frequently highlight the risk of anti-sperm antibody production resulting from needle-induced breaches of the blood–testis barrier, these sampling procedures appear to pose no significant harm to testicular tissue. Moreover, they offer critical diagnostic value in cases where US imaging fails to reveal abnormalities, and hormonal profiles remain within normal ranges [115,138,139,140]. Treatment depends on the cause of the orchitis. If an infectious agent has been detected, a specific antibiotic therapy is conducted. NSAIDs are also commonly used in association with local cold therapy (cold packing of the scrotum). If only one testis is affected, hemicastration is also proposed [114].

Finally, scrotal overheating is a possible cause of testis dysfunction. Questions about the dog’s environment, especially his favorite resting place, should be asked if orchitis is suspected.

#### 4.2.2. Infectious Causes

Common infectious diseases will often lead to orchitis or prostatitis and not only reduce semen quality. The most common infectious agents found in prostatic syndrome and orchitis are bacteria (*Escherichia coli*, *Klebsiella* spp., *Pseudomonas* spp., *Staphylococcus* spp., *Streptococcus* spp., *Brucella* spp., *Mycoplasma* spp., *Ureaplasma* spp., rare cases of Leishmania have been reported), but also fungal organisms such as *Blastomyces dermatitidis*, *Cryptococcus neoformans*, and *Coccidioides immitis* [114,115].

Semen culture should always be conducted, especially when cytology of the semen reveals the presence of inflammation (presence of inflammatory cells) and bacteria. It is important to take clinical aspects into account when interpreting culture results since normospermic dogs’ cultures can be positive [141]. All three fractions can be contaminated, but the first fraction is physiologically more impacted, and cultures should be conducted on the second or the third fraction [142,143]. In humans, the semen microbiome is well studied, and a link has been established between some bacterial populations and semen quality [144].

*Mycoplasma* remains a major concern among breeders, who frequently attribute infertility cases to its presence. However, current research identifies it as a commensal bacterium within the male genital tract, commonly colonizing the prepuce and sperm-rich fraction of healthy, normofertile stud dogs without causing any alterations [145,146]. While the pathogenic potential of *Mycoplasma* spp. is recognized, its direct impact on male fertility remains ambiguous. Consequently, PCR results from semen samples require careful interpretation to avoid overdiagnosis, and a comprehensive judgment should be made in combination with clinical symptoms and cytological results [145,146].

In contrast, *Brucella canis* demands heightened vigilance due to its proven role in infertility across both male and female dogs, compounded by its zoonotic risk. This pathogen can induce orchitis, epididymitis, prostatitis, endocarditis, uveitis, and discospondylitis in males. Semen quality may initially appear unaffected, but characteristic morphological defects—such as head-to-head sperm agglutination along with bent tails, double tails, swollen midpieces, retained protoplasmic droplets, and deformed acrosomes—can manifest within two weeks post-infection. Notably, fluctuating semen quality (e.g., normal results followed by abrupt abnormalities over weeks) should raise suspicion of *B. canis* infection [112,113]. Suspected cases warrant a minimum of PCR testing on both semen and seminal fluid, paired with serological analysis to confirm diagnosis [112].

#### 4.2.3. Hormonal Tests

Several hormones are commonly measured to assess genital function.

Testosterone is typically the first hormone assessed in clinical practice, though results require cautious interpretation. Its production exhibits diurnal fluctuations, and a single blood assay may yield misleading results due to this inherent variability [147,148]. In a study comparing basal levels of testosterone between fertile and infertile dogs, lower concentrations were found in the infertile group, but these levels remained within the normal range. This shows how difficult it is to interpret isolated measurements in individual patients, as a single value without additional context does not provide diagnostic certainty [149].

Estrogens are derived from testosterone via aromatization. They generally remain low in male dogs—even during stimulation tests. Despite their low concentrations, estrogens play a critical role in male canine reproductive physiology. They directly influence testicular and epididymal function and modulate the hypothalamic–pituitary axis (HPA) through negative feedback, underscoring their regulatory importance [150].

Prolactin is easily measurable in routine practice, but its role in fertility in dogs is still poorly understood compared to humans. In humans, hyperprolactinemia is associated with decreased libido, erectile dysfunction, and ejaculatory dysfunction [151]. In dogs, however, studies show that suppression of prolactin with cabergoline does not significantly impair semen quality [152]. Nevertheless, in the author’s opinion, the determination of prolactin as part of a comprehensive endocrine examination is still important, especially in the investigation of multifactorial reproductive disorders.

Finally, anti-Müllerian hormone (AMH) produced by Sertoli cells is being increasingly studied in males and females, particularly as a marker for assessing gonadal function. In male dogs, AMH levels appear to be negatively correlated with sperm morphology as well as total and progressive mobility. Therefore, AMH concentrations may serve as a prognostic indicator of fertility in stud dogs. However, further research is required to confirm these findings [116,153].

As explained earlier, single values of testosterone or estrogens usually do not give us conclusive results. A stimulation test using the stimulation function of hypothalamic pituitary hormones (GnRH and LH) can be used to assess endocrine function. hCG, which has an LH-like action in the dog, is commonly used in practice. Different doses are available; the author uses hCG at a dose of 50 IU/kg intramuscular. The first blood sample is taken before hCG injection, and the second one can be taken between 60 and 120 min after injection [115,117,154]. Table 3 gives a few possible interpretations of the hormonal values obtained (according to different studies and the review of Mason, 2023) [115].

#### 4.2.4. Nutrition

The direct impact of nutrition on fertility is scarcely described in both males and females. The presence of phytoestrogens in humans and animal food is regularly questioned. In males, the consumption of phytoestrogens appears to negatively affect fertility by altering both testicular anatomy—e.g., reducing the number of seminiferous tubules and the thickness of the spermatogenic epithelium—and testicular function, resulting in an increased number of abnormal spermatozoa and a lower sperm concentration in the ejaculate [115,155]. Another common problem is the use of bones and raw food (BARF). While the improper storage of such foods poses a clear risk of infectious diseases, their nutritional adequacy is also frequently questioned. Furthermore, a direct negative influence on semen quality is suspected [156].

Nutritional supplementation to enhance fertility is more documented. Table 4 summarizes the data found in the literature to propose efficient supplementation to improve semen quality and fertility.

### 4.3. Decision Tree

**Figure 5:**
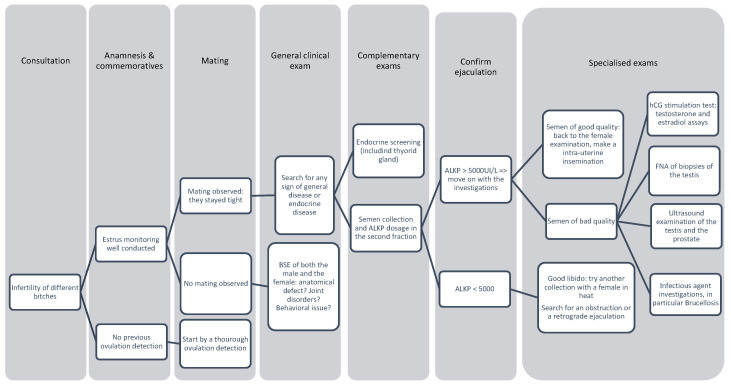
Decision Tree for the Management of a Stud Dog Presented for Infertility. This algorithm reflects the authors’ routine clinical approach and does not represent a universally accepted consensus.

Here is a proposition of a decision tree concerning the management of a stud dog presented for infertility. This is what the authors follow in their common practice, and not a consensual point of view (Figure 5).

The development of this decision tree follows the key points outlined in the Section 4. The initial part of the consultation focuses on taking a thorough anamnesis, including a review of the stud dog’s medical history and previous matings, with particular attention to how those matings were managed. A BSE is then mandatory for both male and female dogs. If the BSE results are normal, further complementary tests may be necessary. The first step involves a standard semen analysis. In cases of oligozoospermia or azoospermia, confirmation of successful ejaculation is performed. If ejaculation is confirmed, more specialized diagnostic tests can be pursued as detailed in the decision tree.

## 5. Conclusions

Bitch fertility is a complex, multifactorial problem that requires comprehensive breeding management and a thorough evaluation of the reproductive health of both the bitch and the dog. A detailed reproductive history in conjunction with a systematic breeding soundness examination (BSE) of both partners forms the basis for successful conception. This review focuses specifically on preconception causes of infertility, which should be systematically ruled out in any fertility workup. The exact timing of ovulation remains crucial and is supported by reliable diagnostic tools such as serial progesterone tests, vaginal cytology and transabdominal or transrectal ultrasound examinations. In addition, new biomarkers such as anti-Müllerian hormone (AMH) offer valuable insights into ovarian reserve and reproductive status. In males, hormone profiling—including testosterone and LH levels—together with semen analysis, is an essential part of the diagnosis. Implementing evidence-based reproductive strategies based on these diagnostic tools facilitates clinical decision-making and improves breeding outcomes. Once the preconceptual factors are clarified, it is equally important to investigate the post-conceptual causes, such as early embryo loss and maintenance of pregnancy, to optimize fertility management.

A holistic understanding and targeted management of each phase of the reproductive cycle is crucial to drive fertility success in canine practice and to support both veterinarians and breeders in achieving optimal reproductive performance.

## Figures and Tables

**Table 1:** Progesterone threshold values using different devices with different dosage methods.

	Elecsys^®^ (Roche, Basel, Switzerland)	Minividas^®^ (Biomérieux, Marcy-l’Étoile, France)	Speedreader^®^ (Virbac, Carros, France)	AU10V^®^ (Fujifilm, Tokyo, Japan)	Catalyst^®^ (IDEXX, Westbrook, ME, USA)	AIA 360^®^ (Kitvia, South San Francisco, CA, USA)
Basal values	<2 ng/mL	<3 ng/mL	<2 ng/mL	<2 ng/mL	<1.9 ng/mL	<2 ng/mL
LH surge	2 ng/mL	3–6 ng/mL	2–4 ng/mL	2–4 ng/mL	2–3 ng/mL	2–4 ng/mL
Ovulation	5 ng/mL	10–12 ng/mL	5–10 ng/mL	4–8 ng/mL	5–12 ng/mL	5–10 ng/mL

**Table 2:** Examples from the literature on whelping rates using artificial insemination with different semen types (fresh, chilled, and frozen).

Reference	Type of Semen Used	Timing (Days Post-Ovulation)	Number of Bitches Inseminated	Pregnancy Rate
**Hollinshead & Hallon, 2017 [69]**	Frozen	3rd and/or 4th	645	71% (and smaller litter size)
	Fresh	2nd, 3rd or 4th	543	80%
	Chilled		15	/
**Thomassen et al., 2006 [68]**	Frozen intra-uterine	2nd and/or 3rd	665	75%
	Frozen intra-vaginal		20	10%
**Linde-Forsberg et al., 1999 [70]**	Frozen intra-uterine	Between the 2nd and 5th	167	84.4%
	Frozen intra-vaginal		141	58.9%

Legend: / no data are available.

**Table 3:** Interpretation of endocrine basal levels and variations after hCG stimulation test (according to Mason, 2023) [115]. T0 is the time of first blood sample, and T1 is 60 to 120 min after the hCG injection.

Results	Possible Interpretations
Low testosterone and estrogen T0 and T1	Primary problem of the HPA axis Testis’ receptors issue
High estrogen T0 and T1	Exogenous estrogen source, including exposure to hormone replacement therapy medications from owners Testicular tumor secreting estrogens Diet phytoestrogen
Estrogens evolving like testosterone or low testosterone with high estrogens at T1	Increased conversion of testosterone into estrogen by an increased activity of aromatase. Therapy proposed: anti-aromatase treatment with anastrazole 1 mg/dog/day for at least a full sperm cycle (at least 2 months)
Prolactin elevated	Therapy proposed: cabergoline 5 μg/kg/d

**Table 4:** The role of key nutrients and supplements in male dog fertility.

Molecule	Effects	Dose	References
Zinc	Prevention of chromatin decondensation. Positive effects on sperm motility. Antioxidant activity on the semen.	3 mg/kg/d	[115,157]
Carnitine	Antioxidant	50 mg/kg/d	[115]
Fish oil (omega-3 and omega-6 supplementation)	Improvement in sperm counts, motility, sperm viability, and fertility. Increase in testicular blood flow.	25% docosahexaenoic acid and 10% eicosapentaenoic acid 54 mg/kg/d	[74,158,159]
Vitamin E and Selenium	Antioxidant: protects the testis from oxidative damage and stabilizes sperm membranes. Improvement in motility and associated fertility.	Vitamin E: 5 mg/kg/d Selenium: 0.6 mg/kg	[160]

## Data Availability

No new data were created or analyzed in this study. Data sharing is not applicable to this article.
